# A Study on the Influence of Gypsum and Ca(OH)_2_ on the Mechanical Properties and Hydration Behavior of Multi-Component Solid Waste-Based Cementitious Materials

**DOI:** 10.3390/ma18091964

**Published:** 2025-04-25

**Authors:** Jiansheng Gou, Shuangxi Li, Chunmeng Jiang, Zhantao Li, Guanglang You

**Affiliations:** 1 College of Hydraulic and Civil Engineering, Xinjiang Agricultural University, Urumqi 830052, China; 17339855797@163.com (J.G.); jiangcm@xjau.edu.cn (C.J.); lizhantao_666@163.com (Z.L.); 13281625037@163.com (G.Y.); 2 Xinjiang Key Laboratory of Hydraulic Engineering Security and Water Disasters Prevention, Urumqi 830052, China

**Keywords:** mechanical property, compressive strength, hydration product, hydration behavior

## Abstract

This study investigates the influence of gypsum (CaSO_4_·2H_2_O) and calcium hydroxide (Ca(OH)_2_) on the hydration behavior and mechanical properties of multi-component solid waste-based cementitious materials. The evolution of hydration products was examined by evaluating compressive strength variations at different dosages and employing advanced analytical techniques, including X-ray diffraction (XRD), Fourier transform infrared spectroscopy (FTIR), thermogravimetric–differential thermogravimetric analysis (TG-DTG), and scanning electron microscopy (SEM). The findings reveal that gypsum addition facilitates the formation of ettringite (AFt), while Ca(OH)_2_ enhances system alkalinity, promoting the precipitation and solidification of calcium silicate hydrate (C-S-H) gel. A balanced incorporation of gypsum and Ca(OH)_2_ significantly improves the early compressive strength and stabilizes hydration products. This study provides theoretical guidance for the effective utilization of solid waste resources and the optimized design of cementitious materials.

## 1. Introduction

With the rapid acceleration of global industrialization and the continuous progression of urbanization, the annual generation of industrial solid waste and construction debris has been increasing significantly, imposing substantial pressure on the environment. In China alone, statistics indicate that the total annual output of industrial solid waste has surpassed 3 billion tons. Among these, typical industrial by-products—such as granulated blast furnace slag (GGBS), fly ash (FA), and silica fume—exhibit high potential for resource utilization. However, their recycling rates remain below 70%. The excessive stockpiling of these materials not only occupies vast land areas but also poses serious environmental risks, including dust emissions, groundwater contamination, and other ecological threats [1,2,3]. Against this backdrop, the development of low-carbon cementitious material systems utilizing industrial solid waste as primary raw materials has emerged as a pivotal strategy to address the environmental challenges of the traditional construction materials industry. This approach not only facilitates the large-scale consumption of solid waste and cost reduction but also significantly curtails carbon emissions, offering substantial environmental and economic benefits.

In recent years, increasing attention has been directed toward the development of cementitious materials derived from multifunctional solid wastes. The term “multi-dimensional solid waste cementitious materials” refers to a new class of binders formulated through physical or chemical activation of industrial solid wastes—such as fly ash, slag, and silica fume—as primary raw materials [4]. Slag, a by-product generated during iron and steel smelting, is predominantly composed of silicates and aluminates with an amorphous glassy structure, exhibiting high latent hydraulic reactivity. Fly ash, the principal solid waste from coal-fired power plants, mainly consists of glassy silica and alumina. Although it possesses some pozzolanic activity, its low reactivity often necessitates activation. Silica fume, an ultrafine powder produced during the smelting of silicon metals and alloys, is characterized by an extremely high specific surface area and a high content of reactive SiO_2_, which can significantly enhance hydration reactions. The nanoscale particles of silica fume are capable of filling the micron-sized voids in slag and fly ash matrices, thereby contributing to a denser microstructure. Additionally, the high reactivity of SiO_2_ in silica fume can accelerate the hydration of low-calcium systems, producing a synergistic effect when combined with other solid wastes [2,5]. Although silica fume possesses considerable economic value, it remains fundamentally an industrial by-product. The environmental benefits associated with its utilization—such as reduced stockpiling and minimized ecological risks—can be further enhanced by partially or wholly replacing traditional cement with solid waste-based cementitious materials. Conventional cement production is characterized by high energy consumption and significant carbon emissions. In contrast, cementitious systems based on multifaceted solid wastes offer a promising alternative, owing to their low-carbon footprint, environmental sustainability, and efficient resource utilization [6]. When appropriately proportioned and activated, solid wastes such as slag, fly ash, and silica fume can be engineered into systems with excellent binding properties, making them suitable for a wide range of applications in civil engineering, transportation infrastructure, and construction materials. Investigating the hydration behavior of these systems is not only essential for optimizing their performance but also provides a scientific foundation for the future development of green and sustainable building materials [7].

Gypsum and calcium hydroxide (Ca(OH)_2_), as commonly used sulfate and alkaline activators, respectively, are widely applied in the development of cementitious materials. Gypsum-based activators promote the formation of hydration products such as ettringite (and occasionally calcite), which significantly enhance the early-age strength of the material. In combination with calcium hydroxide and sodium aluminosilicates, gypsum contributes to the formation of a more complex and stable cementitious microstructure [8]. Calcium hydroxide, serving as an alkaline activator, creates a highly alkaline environment that facilitates the hydration of reactive solid wastes such as slag and fly ash, thereby improving the mechanical performance of the resulting binders [9]. The incorporation of both gypsum and Ca(OH)_2_ into multifunctional solid waste-based cementitious systems not only optimizes material composition but also leverages the synergistic interaction between sulfate and alkaline activation, further enhancing the overall performance of the material.

At present, the engineering application of multifunctional solid waste-based cementitious materials continues to face several technical challenges, including a slow hydration rate, inadequate early strength development, and uncertainties regarding long-term durability and stability [10]. Consequently, it is essential to investigate the hydration mechanisms and microstructural evolution of these materials in order to optimize mix designs and improve their engineering adaptability. Extensive research, both domestic and international, has focused on the role of gypsum and calcium hydroxide (Ca(OH)_2_) in enhancing the performance of cementitious systems. Studies have demonstrated that the incorporation of gypsum can significantly improve the early-age strength of slag-based binders and enhance their resistance to cracking [11]. In contrast, the addition of Ca(OH)_2_ can activate the latent reactivity of fly ash and slag, promoting the formation of hydration products and thereby contributing to the long-term strength development of the material [12]. Yanning et al. investigated the effects of calcium hydroxide (Ca(OH)_2_) as an alkaline activator on the workability and mechanical properties of a composite cementitious system comprising nickel slag, iron tailings slag (FETNS), and ordinary Portland cement (OPC). The results indicated that the addition of Ca(OH)_2_ reduced the initial setting time of the system by approximately 40% and the final setting time by about 20%. Furthermore, the compressive strengths at 3, 7, 28, and 60 days increased by 51.95%, 45.27%, 8.53%, and 8.9%, respectively [13]. Similarly, Yunpeng et al. studied the mechanical behavior of cementitious composites incorporating high volumes of fly ash and reinforced with polyvinyl alcohol (PVA) fibers, using calcium sulfate as an activating agent. Their findings revealed that calcium sulfate exhibited a certain degree of activation, promoting the formation of calcite. The early-age strength of the PVA-engineered cementitious composites (PVA-ECC) was enhanced, while the tensile strain capacity decreased by less than 0.92% [14]. Zeng Liang et al. demonstrated that in the presence of desulfurization gypsum and alkali, the hydration of various material components and slag was mutually enhanced. The primary early hydration products were ettringite (AFt) and calcium silicate hydrate (C-S-H) gel, with an increasing accumulation of hydration products over time. Needle-like AFt crystals embedded within the C-S-H gel contributed to a denser hardened paste structure, resulting in a gradual increase in strength [15]. Additionally, research by Ni Zhenkun et al. indicated that the incorporation of desulfurization gypsum effectively prolonged the setting time of alkali-activated cementitious materials, with its dosage being positively correlated with setting time but negatively correlated with strength, hydration rate, and cumulative heat release [16]. However, there remains a lack of systematic research on the synergistic effects of gypsum and Ca(OH)_2_ on the mechanical properties and hydration behavior of multi-component solid waste-based cementitious materials.

Furthermore, the hydration behavior of multi-component solid waste-based cementitious materials is a complex physicochemical process involving the interactions of various solid waste components and the formation and evolution of hydration products. Li L. et al. examined the influence of four alkali-sulfate activators on the hydration behavior of blast furnace slag. Their findings demonstrated that sodium sulfate, in particular, significantly accelerated hydration kinetics and shortened setting times. This enhancement was attributed to the higher pH of the pore solution, which facilitated a faster precipitation rate and promoted the transition from initial solidification to the formation of long-term C-A-S-H gel phases [17]. Valentino et al. employed nuclear magnetic resonance (NMR), X-ray diffraction (XRD), and thermogravimetric analysis (TGA) to investigate the combined effects of hard gypsum and limestone on the hydration of tricalcium aluminate (C_3_A). The results revealed that the identity and quantity of the AFm phases formed were highly dependent on the nature of the available anionic species, highlighting the sensitivity of C_3_A hydration to the chemical environment [18]. Yang Li explored the synergistic activation of slag using a combination of calcium hydroxide and sodium sulfate for the preparation of alkali-activated cementitious materials. The study analyzed the influence of varying the proportion of these activators on hydration behavior. The results indicated that the combined use of calcium hydroxide and sodium sulfate provided a superior activation effect, promoting the formation of a denser gel matrix and reducing the occurrence of microcracks, as evidenced by microstructural observations [19]. By analyzing the effects of red mud, fly ash, desulfurization gypsum, and cement on the heat release rate, total heat release, hydration process, and hydration kinetics, researchers have explored their roles in the hydration mechanisms of cementitious materials, ultimately clarifying the overall hydration process [20]. Zhang Yichao et al. investigated low-carbon concrete with phosphogypsum-based cementitious materials and recycled aggregates as primary raw materials, concluding that the interfacial transition zone (ITZ) is the key factor influencing microscopic damage characteristics [21]. Qing L et al. utilized colloidal nano-silica (CNS) as an additive to enhance the strength of CaO/CaSO_4_^−^ activated slag cementitious materials, studying its effects on workability, hydration kinetics, hydration product composition, polymerization degree, and microstructure [22]. However, the underlying mechanisms governing the influence of gypsum and Ca(OH)_2_ on the hydration behavior of multi-component solid waste-based cementitious materials—particularly their role in regulating the composition and microstructure of hydration products—remain to be further investigated.

This study aims to investigate the effects of gypsum and calcium hydroxide (Ca(OH)_2_) on the mechanical properties and hydration behavior of multi-component solid waste-based cementitious materials and to elucidate their underlying mechanisms. Using cement, fly ash, slag, and silica fume as the primary components, the content, proportion, and interactions of gypsum and Ca(OH)_2_ within the multi-component system were systematically examined. The study analyzed their influence on compressive strength, flexural strength, hydration product composition, and microstructure, providing a theoretical foundation and technical support for the optimized design and performance enhancement of these materials. The findings of this research will contribute to advancing solid waste utilization technologies and fostering the green transformation of the construction industry.

## 2. Test

### 2.1. Test Raw Materials

In this study, P·O 42.5 R ordinary Portland cement, sourced from Tianshan Cement Plant in Xinjiang, was used. The slag, fly ash, and silica fume were obtained from S75 granulated blast furnace slag produced by Xinjiang Baoxin Shengyuan Company (Urumqi, China) and Class II F fly ash. Their chemical compositions are detailed in Table 1. Gypsum and calcium hydroxide, both of analytical grade, were purchased from the Urumqi chemical market in Xinjiang. The fine aggregate used was continuously graded natural sand, with a fineness modulus of 2.92 and an apparent density of 2597.4 kg/m^3^. A polycarboxylate superplasticizer was used as the water reducer, while laboratory tap water served as the mixing water.

### 2.2. Test Methods

According to Zhang Bowen [23], the mass ratio of slag to fly ash was set at 7:3, with a silica fume content of 3% and a cement content of 20%. The water–binder ratio was maintained at 0.5, while the solid gypsum content was fixed at 10%. Additionally, calcium hydroxide was introduced as an activator, with its content ranging from 2% to 10%. The mixing ratios illustrating the effect of gypsum content on multi-component solid waste-based cementitious materials are presented in Table 2, while the mixing ratios for gypsum combined with calcium hydroxide are detailed in Table 3.

#### 2.2.1. Mechanical Performance Test

The compressive and flexural strength tests were conducted in accordance with the GB/T 17671-2021 Cement Mortar Strength Test Method (ISO method). For each mix ratio, three 40 mm × 40 mm × 160 mm specimens were prepared. The specimens were cured in a standard constant-temperature curing room at (20 ± 2) °C and 95% relative humidity for 24 h, followed by demolding. They were then placed back in the curing room until the designated testing age. The compressive and flexural strength tests were performed using a YAW-300D cement mortar compressive and flexural testing machine [24].

#### 2.2.2. XRD

After the compressive strength test, the central portion of the sample was immersed in anhydrous ethanol for three days to halt hydration. The fragments were then ground using a grinding apparatus, and the resulting powder was dried in a blast drying oven at 45 °C for 1–4 h. The mineral composition of the hydration products in the multi-component solid waste-based cementitious material was analyzed using X-ray diffraction (XRD). The powder samples were tested within a 2θ angle range of 5–80° using a Rigaku Ultima IV X-ray diffractometer (XRD, copper radiation after nickel filtration) from Akishima, Japan. The scanning rate and step size were set to 2° per minute and 0.02°, respectively.

#### 2.2.3. FTIR

The FTIR test was performed using a Fourier transform infrared spectrometer of Tianguang FTIR-960 (Tianguang Instrument, Beijing, China). After the specimen reaches the curing age, the cement stone sample that is not in contact with the surface is broken, and the sample is dried to constant weight at 50 °C in vacuum. The functional groups and chemical bonds of the sample are detected, and the KBr tableting method is used. The test range is 4000~400 cm^−1^.

#### 2.2.4. TG-DTG

The thermal weight loss curve of the test block powder sample was determined by simultaneous thermal analyzer (TG-DTG, PerkinElmer Pyris1, PerkinElmer, Waltham, MA, USA), which was used to calculate the content of crystal water and calcium hydroxide in the hydration product. The test atmosphere was air, the temperature range was 25~1000 °C, the heating rate was 10 °C/min, and the purge and protective gas N_2_ rate was 20 mL/min.

#### 2.2.5. SEM

The hydration products of multi-component solid waste cementitious material paste were characterized by scanning electron microscopy (SEM). The compressive test broken sample sheet was selected. Before the test, the sample was dried at 50 °C until constant weight, and the surface of the sample was sprayed with gold. The microstructure of the hydration products was observed by an ultra-plus scanning electron microscope from Germany under the 10 kV accelerated voltage.

## 3. Result and Discussion

### 3.1. Effect of Gypsum on Strength of Multiple Solid Waste Cementitious Materials

**Figure 1:**
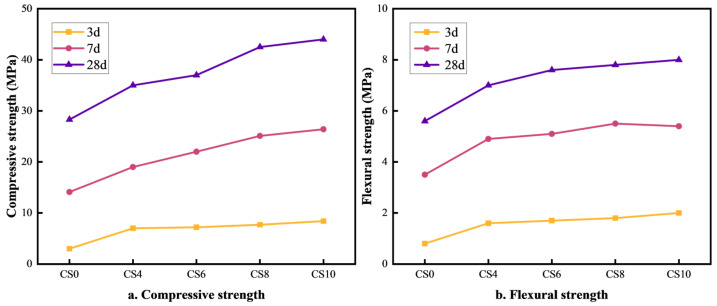
Effect of gypsum content on compressive and flexural strength of cementitious materials.

The effect of gypsum content on the strength of multi-component solid waste-based cementitious materials is illustrated in Figure 1a,b. As seen in Figure 1a, when the gypsum content increases from 4% to 10%, the compressive strength of the cementitious material also increases. The optimal compressive strength is achieved at a 10% gypsum content, with the CS10 group reaching 8.4 MPa, 26.4 MPa, and 44 MPa at 3 days (3d), 7 days (7d), and 28 days (28d), respectively. Compared to the CS0 group (without gypsum), the compressive strength of CS10 was 5.4 MPa, 12.3 MPa, and 15.7 MPa higher at 3d, 7d, and 28d, respectively. This enhancement is attributed to the SO_4_^2−^ ions from gypsum, which react with the tricalcium aluminate (C_3_A) phases in cement, slag, and fly ash to form ettringite (AFt). The formation of AFt densifies the structure and enhances compressive strength. Additionally, an appropriate gypsum content regulates the hydration rate, delays the heat of hydration release, and optimizes structural densification, contributing to increased compressive strength [25]. As shown in Figure 1b, the flexural strength also increases as the gypsum content rises from 4% to 10%, with the highest strength achieved at 10% gypsum content. The flexural strength values for CS10 at 3d, 7d, and 28d are 2 MPa, 5.5 MPa, and 8 MPa, respectively. Compared to the CS0 group, the CS10 flexural strength was 0.2 MPa, 1.9 MPa, and 2.4 MPa higher at 3d, 7d, and 28d, respectively. This improvement is due to the enhanced formation of AFt (calcium alumina) and AFm (calcium aluminate hydrate), which improve the material’s toughness and flexural capacity. Gypsum also helps fill pores, reduce crack expansion, improve toughness, and enhance flexural strength [26]. Overall, the incorporation of gypsum contributes to the long-term strength development of multi-component solid waste-based cementitious materials, allowing them to meet the strength requirements of 42.5-grade Portland cement.

### 3.2. The Effect of Gypsum Mixed with Calcium Hydroxide on the Strength of Multi-Component Solid Waste Cementitious Materials

**Figure 2:**
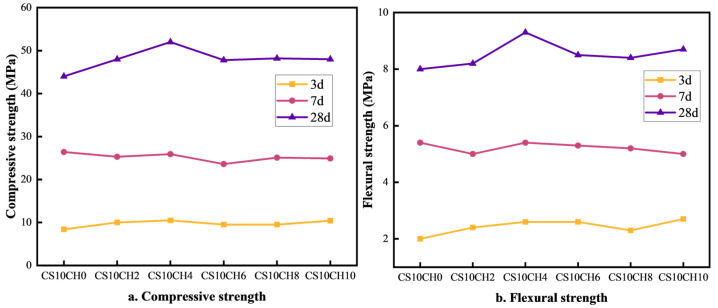
Effect of gypsum mixed with calcium hydroxide on compressive and flexural strength of cementitious materials.

The effect of gypsum mixed with calcium hydroxide (Ca(OH)_2_) on the strength of multi-component solid waste cementitious materials is illustrated in Figure 2a,b. As observed, when Ca(OH)_2_ is introduced into a system with a single gypsum activator, the overall strength first increases and then decreases. Compared to the CS10CH0 group (with gypsum but without Ca(OH)_2_), the addition of Ca(OH)_2_ initially enhances both compressive and flexural strength. The CS10CH4 group exhibits the highest strength values, with compressive strengths of 10.5 MPa, 25.9 MPa, and 52.0 MPa at 3 days (3d), 7 days (7d), and 28 days (28d), respectively, and flexural strengths of 2.6 MPa, 5.4 MPa, and 9.3 MPa at the same time points. While the 3d and 7d strength changes are relatively small, the 28d compressive strength increases by 8.0 MPa, and the flexural strength increases by 1.5 MPa compared to the CS10CH0 group. Gypsum, as a slow-hydrating material, reacts with cement and hydration products to increase the formation of cementitious compounds, while Ca(OH)_2_ facilitates the formation of calcium–silicate–hydrate (C-S-H) gel, thereby enhancing strength. In the CS10CH4 group, a moderate amount of Ca(OH)_2_ significantly enhances the hydration reaction, improves the compactness of hydration products, and strengthens both compressive and flexural properties. However, excessive Ca(OH)_2_ may lead to overly rapid hydration or incomplete reactions, resulting in a more fragile structure and a decline in compressive strength. Therefore, an optimal amount of Ca(OH)_2_ plays a crucial role in improving the long-term strength of the material. Gypsum releases SO_4_^2−^ and Ca^2+^, which react with [Al(OH)_4_]^−^ and Ca^2+^ to form calcite, while Ca(OH)_2_ provides OH^−^ ions, promoting the formation of C-S-H gel in solution. This reinforces the internal structure of the cementitious material [19,27,28]. Thus, the synergistic activation of gypsum and Ca(OH)_2_ is more effective in enhancing the compressive strength of multi-component solid waste cementitious materials compared to gypsum alone.

### 3.3. XRD Analysis

**Figure 3:**
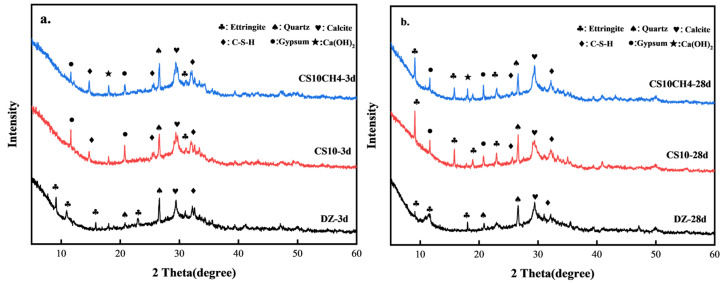
XRD spectra of solid waste cementitious materials. (**a**) 3d (**b**) 28d.

Figure 3 presents the XRD patterns of the CS10 and CS10CH4 groups, along with the control group, at 3 days (3d) and 28 days (28d). From Figure 3a,b, it can be observed that the hydration products in the DZ group mainly include calcium–silicate–hydrate (C-S-H), quartz (SiO_2_), calcite (CaCO_3_), and ettringite (AFt). Upon incorporating gypsum and calcium hydroxide, additional hydration products such as unhydrated gypsum and Ca(OH)_2_ appeared. In the DZ group, a strong calcite peak is already visible at 3d, indicating that calcite forms early in the hydration process. However, by 28d, the calcite peak weakens, suggesting that some calcite transforms into more stable hydration products. The peaks of quartz and calcite remain relatively unchanged, implying that these inert materials remain stable and do not actively participate in hydration. In the CS10 group, a small amount of AFt is generated at 3d, due to the reaction between gypsum and calcium aluminate phases in cement, forming AFt. However, the AFt peak remains weak, indicating that AFt production is still limited in the early hydration stage. A pronounced gypsum peak at 3d suggests that gypsum is not yet fully converted into AFt and other hydration products. The formation of C-S-H continues, while quartz and calcite remain stable. By 28d, the production of AFt significantly increases as more gypsum transforms into AFt. The C-S-H peak intensifies, indicating that gypsum enhances the hydration reaction. Meanwhile, the gypsum peak diminishes, confirming its consumption and conversion into AFt or other hydration products [29]. In the CS10CH4 group, at 3d, a small amount of AFt is still formed, although the addition of calcium hydroxide appears to slightly delay AFt formation. The AFt peak remains weak, suggesting that Ca(OH)_2_ may inhibit AFt production. Similar to the other groups, quartz and calcite remain stable, while C-S-H continues to be the primary hydration product. A significant Ca(OH)_2_ peak appears at 3d, indicating that Ca(OH)_2_ participates in the hydration reaction. The strong gypsum peak suggests that not all gypsum has been consumed, with some still undergoing conversion to AFt. By 28d, AFt production increases, and gypsum releases SO_4_^2−^ and Ca^2+^, which react with [Al(OH)_4_]^−^ and Ca^2+^ to form calomel. This process strengthens the cementitious system structure, thereby enhancing mechanical strength. The Ca(OH)_2_ peak decreases, likely due to its reaction with other components to form C-S-H and additional hydration products. The weakening gypsum peak confirms its full participation in hydration reactions [30].

### 3.4. FTIR Analysis

**Figure 4:**
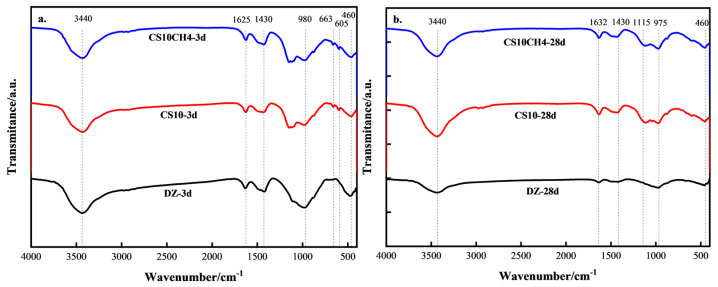
FTIR spectra of solid waste cementitious materials. (**a**) 3d (**b**) 28d.

Figure 4 presents the FTIR spectra of the CS10 and CS10CH4 groups, along with the control group, at 3 days (3d) and 28 days (28d). At approximately 3440 cm^−1^, the hydroxyl characteristic absorption band is observed, which corresponds to the O-H stretching vibration in the C-S-H hydration product. This peak is present in all groups at both 3d and 28d, confirming the formation of C-S-H and calcium hydroxide (CH) as hydration products. The O-H vibrational peak is stronger in the CS10CH4 group, which may indicate a higher degree of C-S-H hydration or an increase in CH production. At around 1632 cm^−1^, a peak corresponding to H-O-H vibration appears, indicating bending vibrations of bound water, which further supports the presence of C-S-H gel. The peak at 1430 cm^−1^ is attributed to the CO_3_^2−^ vibration, signifying that the specimen was exposed to CO_2_ from the air, leading to carbonation. This peak is more prominent in the CS10CH4 group, suggesting that the combination of gypsum and calcium hydroxide enhances the generation of CH, which, in turn, leads to more CaCO_3_ formation. At around 1115 cm^−1^, the SO_4_^2−^ stretching vibration is observed, with its intensity decreasing at 28d. This reduction indicates that some of the SO_4_^2−^ has reacted during hydration to form calomelite. The peak at approximately 980 cm^−1^ is related to the asymmetric stretching vibration of the Si-O bond, signaling the formation of C-S-H gel. This peak intensifies at 28d, indicating continued development of C-S-H as hydration progresses. The peak at 663 cm^−1^ is associated with the bending vibration of SO_4_^2−^ in hydration products. Since both the CS10 and CS10CH4 groups contain gypsum, the early gypsum dissolution at 3d releases SO_4_^2−^, which reacts with aluminate in the solution to form calomelite (AFt). This results in an enhanced absorption peak at this wavenumber. By 28d, some of the AFt has transformed into monosulfur-type hydrated calcium thioaluminate (AFm), leading to the disappearance of this absorption peak. The absorption peaks between 460 cm^−1^ and 605 cm^−1^ are attributed to Si-O and Al-O vibrations. At 3d, these peaks are primarily associated with the formation of early hydration products such as C-S-H, C-A-S-H, and AFt. By 28d, the C-S-H structure becomes more stable, and C-A-S-H may transform into a more mature C-S-H, resulting in a weakening of the Si-O and Al-O absorption peaks. The presence of calcium hydroxide in the CS10CH4 group may have accelerated the evolution of these structures, leading to the disappearance of the characteristic peaks in this region at 28d [31,32,33].

The CS10CH4 samples exhibited pronounced enhancements in the characteristic C-S-H absorption peak at 980 cm^−1^, as well as in the skeletal vibration peaks at 460 cm^−1^ and 605 cm^−1^, at both 3 and 28 days. These observations suggest a more complete hydration reaction, higher formation of hydration products, and a denser microstructure. Consistent with the spectroscopic results, the compressive strength tests confirmed that the CS10CH4 samples displayed superior mechanical performance across all curing ages, with the 28-day compressive strength reaching approximately 52 MPa, significantly higher than that of other groups.

In comparison, although increasing the gypsum content in the single-gypsum system led to a gradual rise in C-S-H peak intensity and compressive strength, the overall strength remained markedly lower than that achieved with the calcium hydroxide-enhanced system. A comprehensive analysis indicates a strong positive correlation between the enhancement of C-S-H and skeletal structure peaks in FTIR spectra and the corresponding increase in compressive strength. These results highlight the critical role of both the type and quantity of hydration products in governing the mechanical properties of multifunctional solid waste-based cementitious materials.

### 3.5. TG-DTG Analysis

**Figure 5:**
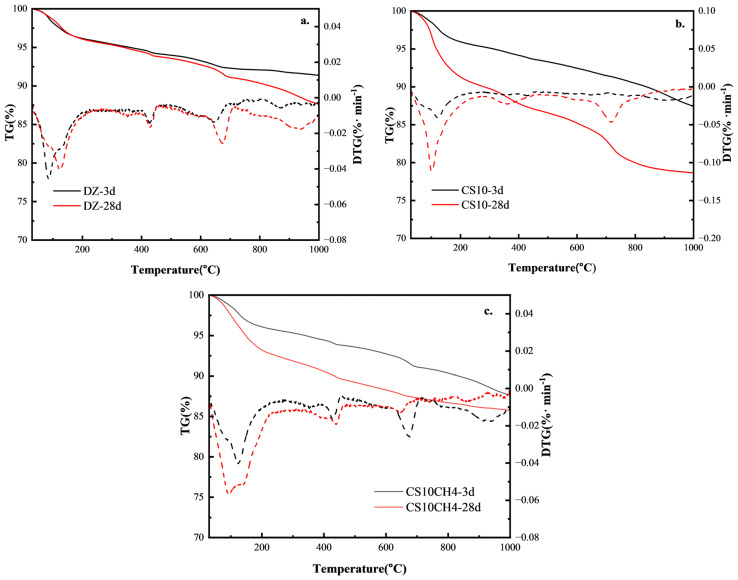
TG-DSC spectra of solid waste gelling materials. (**a**) DZ, (**b**) CS10, (**c**) CS10CH4.

The TG-DSC analysis of the multifunctional solid waste gelling materials reveals insights into their hydration products and aids in understanding the hydration reaction mechanism. Figure 5 shows the TG-DTG curves for the three solid waste-based gelling materials. The thermogravimetric analysis reveals clear weight loss intervals between 50 °C and 1000 °C. The first interval, typically between 50 °C and 200 °C, is primarily caused by the removal of molecularly bound water from calixarenes and C-S-H gels. The second interval, around 480 °C, corresponds to the decomposition of Ca(OH)_2_ into CaO, where a rapid weight loss is observed. The third interval, between 700 °C and 800 °C, is due to the high-temperature decomposition of calcium carbonate in the system [34].

For the control group, at 3d, the TG curve shows minimal mass loss, indicating slow hydration with fewer hydration products. At 28d, the mass loss increases slightly, suggesting that the volcanic ash reaction of fly ash and slag took place later, though the hydration was still limited and not sufficiently activated. When gypsum is added, at 3d, there is a significant increase in mass loss between 100–200 °C, corresponding to the dehydration of calcite (AFt) and C-S-H, indicating that gypsum has activated the aluminum phase and promoted the early hydration reaction. At 28d, the mass loss increases further, showing the further activation of fly ash and slag, an increase in AFt content, and enhanced C-S-H gel structure, improving the densification of the gelation system. For the CS10CH4 group, at 3d, the mass loss between 400–500 °C increases significantly, indicating a higher calcium hydroxide (CH) content in the system and suggesting that the hydration reaction of the cementitious materials has accelerated. At 28d, the mass loss increases further, and the reduction in weight loss between 600–800 °C indicates a more complete volcanic ash reaction, where CH is consumed by silica fume and fly ash to produce more C-S-H gels, contributing to increased strength [35].

According to the DTG curves, it can be observed that in the DZ control group, the weight loss peak between 100–200 °C is weak, indicating a low content of AFt and C-S-H, with limited hydration products. The decomposition peak of CH at 400–500 °C is distinct, but due to under-excitation of the fly ash and slag, less CH is consumed, and the volcanic ash reaction is weaker. The smaller weight loss at 600–800 °C suggests limited carbonate formation and fewer hydration products. In the CS10 group, the weight loss peak at 100–200 °C is significantly enhanced, indicating an increase in AFt content and suggesting that gypsum activates the aluminum phase, increasing the early hydration product formation rate. The weight loss peak of CH at 400–500 °C is smaller, suggesting that CH is consumed by fly ash and slag, enhancing the volcanic ash reaction and promoting the generation of C-S-H. The weight loss between 600–800 °C is slightly higher, indicating some carbonate formation, but still at a low level. In the CS10CH4 group, the weight loss peak at 100–200 °C is further enhanced, indicating the highest AFt and C-S-H content, and demonstrating that the synergy between gypsum and CH accelerates the activation of fly ash and slag, thereby increasing the hydration rate. The CH decomposition peak at 400–500 °C is relatively small, suggesting that CH is consumed in large quantities, enhancing the volcanic ash reaction and promoting the formation of C-S-H. The weight loss between 600–800 °C is the least, indicating more complete hydration with minimal unreacted carbonate in the system, and maximally stimulating the activity of silica fume [36].

A more complete hydration reaction in the multifunctional solid waste-based cementitious materials was associated with lower 28-day mass loss, as evidenced by the TG-DTG analysis. The DTG curves exhibited more pronounced peaks in the ranges of 100–200 °C and 600–800 °C, corresponding to the dehydration of hydration products such as C-S-H gels and ettringite, and the decomposition of calcium carbonate, respectively. These thermal signals reflect the formation of greater quantities of reaction products. When compared with mechanical performance, samples exhibiting higher amounts of hydration products and denser microstructures—such as the CS10CH4 group at 28 days—demonstrated superior compressive strength. This suggests that TG-DTG characteristics are reliable indicators of the degree of hydration and show a strong positive correlation with the mechanical properties of the cementitious system.

### 3.6. SEM Analysis

**Figure 6:**
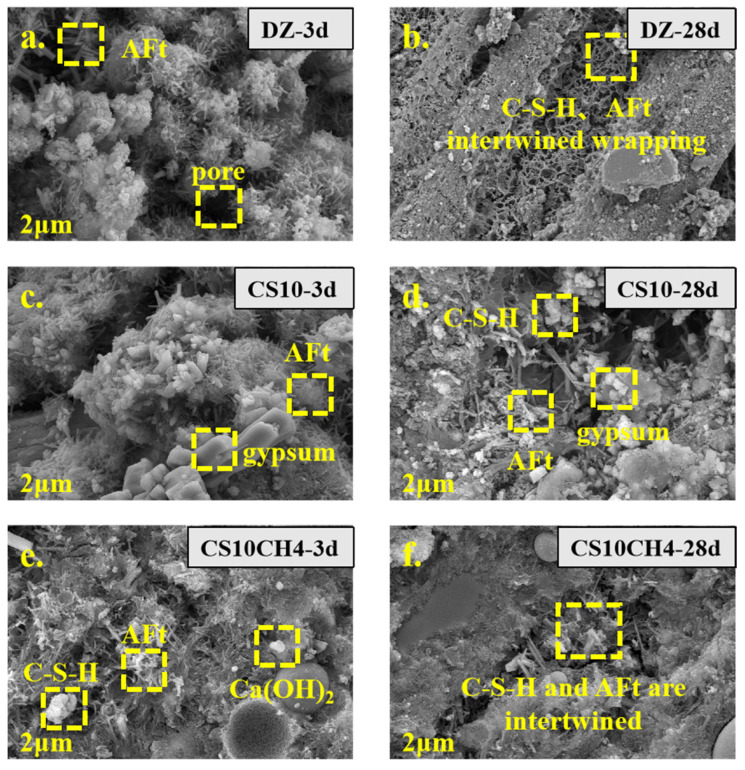
SEM spectra of solid waste gelling materials. (**a**) DZ-3d, (**b**) DZ-28d, (**c**) CS10-3d, (**d**) CS10-28d, (**e**) CS10CH4-3d, (**f**) CS10CH4-28d.

Figure 6 presents the SEM images of solid waste gels with different activators at various ages. The characteristic major products of each age, such as AFt, C-S-H gels, tricalcium silicate, and dicalcium silicate, are clearly visible. In the control group, the needle-and-rod calcium aluminate structures are evident, while the gel-like structures produced by C-S-H and AFt become more apparent as hydration progresses. The SEM image of CS10, with the addition of gypsum, clearly shows prismatic gypsum crystals adhering to the surface of the gel material. These crystals react with tricalcium silicate and dicalcium silicate, inducing the conversion of hydration products towards AFt and C-S-H. In the CS10CH4 group, where Ca(OH)_2_ is added as an activator, the combination of AFt, C-S-H, and Ca(OH)_2_ is visible. The addition of Ca(OH)_2_ seems to accelerate the early hydration process, promoting the formation of more C-S-H. After 28 days, a significant amount of C-S-H is seen tightly encapsulating the material, indicating a rapid hydration process [37].

## 4. Conclusions

(1). The optimal dosage of gypsum in the multifunctional solid waste-based cementitious system was determined to be 10%, at which the compressive strengths at 3, 7, and 28 days reached 8.4 MPa, 26.4 MPa, and 44.0 MPa, respectively. This dosage notably contributed to the enhancement of the later-age strength. Compared with single-gypsum activation, the synergistic activation using a combination of gypsum and calcium hydroxide (Ca(OH)_2_) proved more effective in improving compressive strength. In the CS10CH4 group, the compressive strengths at 3, 7, and 28 days were recorded as 10.5 MPa, 25.9 MPa, and 52.0 MPa, respectively, indicating a significant improvement, particularly in long-term strength development.
(2). The CS10CH4 group exhibited the highest degree of hydration and the greatest formation of C-S-H gels, which significantly contributed to strength development. This improvement is primarily attributed to the additional calcium hydroxide, which enhanced the alkalinity of the system, thereby promoting pozzolanic reactions and increasing the yield of hydration products. Moreover, the elevated alkalinity accelerated the formation of ettringite (AFt), further enhancing the material’s performance. Overall, the combined activation using gypsum and calcium hydroxide effectively facilitated the hydration process of multifunctional solid waste-based cementitious materials, leading to improved mechanical properties and enhanced structural stability.
(3). With the global emphasis on sustainable development and environmental protection, multifunctional solid waste gelling materials are poised to gain new development opportunities. Future research can further explore the synergistic use of gypsum and calcium hydroxide (Ca(OH)_2_) with other activators to optimize the performance of multi-component solid waste cementitious systems. Additionally, integrating big data and artificial intelligence technologies to establish predictive models for material performance can provide a scientific foundation for the optimized design of these materials. Strengthening application-focused research in real-world engineering projects will also be crucial in promoting the large-scale adoption and implementation of such sustainable materials.

## Figures and Tables

**Table 1:** Chemical composition of cementitious materials (%).

Name	CaO	SiO_2_	Al_2_O_3_	SO_3_	MgO	Fe_2_O_3_	TiO_2_	K_2_O	Na_2_O
Cement	63.82	22.18	4.46	2.55	2.42	3.22	0.82	0.04	0.51
Slag	39.30	34.53	12.73	0.40	8.27	1.93	0.90	0.80	0.30
Flyash	4.80	56.20	26.01	1.02	1.56	5.09	1.80	0.90	0.29
Silica fume	0.29	87.50	2.15	0.52	3.86	0.70	1.11	3.31	0.56

**Table 2:** Test mix ratio (g).

No	Gypsum	Water	Slag	Flyash	Silica Fume	Cement	Sand
DZ	0	225	242.55	103.95	13.5	90	1350
CS4	18	221.4					
CS6	27	219.6					
CS8	36	217.8					
CS10	45	216					

**Table 3:** Test mix ratio (g).

No	Ca(OH)_2_	Gypsum	Slag	Flyash	Cement	Silica Fume	Water	Sand
CS10CH0	0	45	242.55	103.95	90	13.5	216	1350
CS10CH2	9							
CS10CH4	18							
CS10CH6	27							
CS10CH8	36							
CS10CH10	45							

## Data Availability

The original contributions presented in this study are included in the article. Further inquiries can be directed to the corresponding author.
